# Review of the genus *Craspedolcus* Enderlein sensu lato in China, with the description of a new genus and four new species (Hymenoptera, Braconidae, Braconinae)

**DOI:** 10.3897/zookeys.647.11247

**Published:** 2017-01-23

**Authors:** Yang Li, Cornelis van Achterberg, Xue-xin Chen

**Affiliations:** 1 State Key Laboratory of Rice Biology and Ministry of Agriculture Key Lab of Agricultural Entomology, Institute of Insect Sciences, Zhejiang University, Hangzhou 310058, China; 2 Shaanxi Key Laboratory for Animal Conservation / Key Laboratory of Resource Biology and Biotechnology in Western China, College of Life Sciences, Northwest University, 229 North Taibai Road, Xi’an, Shaanxi 710069, China

**Keywords:** Hymenoptera, Braconidae, Braconinae, Craspedolcus, Maculibracon, new genus, new species, Oriental, China, Thailand, Vietnam

## Abstract

A new genus is split off the genus *Craspedolcus* Enderlein, 1920 (Hymenoptera, Braconidae, Braconinae): *Maculibracon* **gen. n.** with type species *Maculibracon abruptus* **sp. n.** The genus *Craspedolcus* Enderlein *sensu stricto* is redefined, a key to both genera and to their species in China, Thailand and Vietnam is included. *Craspedolcus obscuriventris* Enderlein, 1920, (**syn. n.**) is a new synonym of *Craspedolcus vagatus* (Smith, 1858), as *Ipobracon maculicosta* Enderlein, 1920 and *Iphiaulax bhotanensis* Cameron, 1907 of *Maculibracon simlaensis* (Cameron, 1899), **comb. n**. The genus *Craspedolcus* is recorded from China for the first time with two species: *Craspedolcus fraternus* Enderlein, 1920, and *Craspedolcus politus* **sp. n.** The genus *Maculibracon* is represented by three species in China: *Maculibracon simlaensis* (Cameron, 1899), **comb. n.** (also present in Vietnam), *Maculibracon hei* **sp. n.** and *Maculibracon luteonervis* **sp. n.** and a fourth species is described from Thailand: *Maculibracon abruptus* **sp. n.** *Hybogaster zebripterae* Wang & Chen, 2008, from China (Fujian) is transferred to *Iphiaulax* Foerster, 1863, (**comb. n.**) and the following names are new combinations in *Maculibracon* **gen. n.**: *Bracon lepcha* Cameron, 1899; *Bracon phaedo* Cameron, 1899; *Bracon simlaensis* Cameron, 1899; *Iphiaulax bhotanensis* Cameron, 1907; *Iphiaulax laertius* Cameron, 1903; *Iphiaulax leptopterus* Cameron, 1903; *Iphiaulax lineaticarinatus* Cameron, 1907; *Ipobracon lissotomus* Roman, 1914; *Ipobracon maculicosta* Enderlein, 1920 and *Iphiaulax pallidicornis* Roman, 1914. *Craspedolcus montezuma* (Cameron, 1887) is provisionally transferred to the genus *Digonogastra* Viereck, 1912.

## Introduction

*Craspedolcus* Enderlein, 1920 (Hymenoptera, Braconidae, Braconinae) is an Oriental and Wallacean genus that neither has been revised nor has its type species fully illustrated. Quicke (1985) redescribed the genus and gives a list of species belonging to it, later augmented by Quicke and van Achterberg (1990). In the Taxapad database 18 species are listed as valid (Yu et al. 2016). The only included New World species, *Craspedolcus montezuma* (Cameron, 1887) from Mexico, was transferred by Enderlein (1920) from *Iphiaulax* Foerster, 1863, to *Craspedolcus* but is here excluded. The short description does not give a clue why it should be included in *Craspedolcus* except for “the centre [of first tergite] with a keel down the middle”. A median keel at the middle of the first tergite occurs also in other genera, and a similar species described in the same paper (*Iphiaulax chontalensis*) has been transferred to *Digonogastra* Viereck, 1912. Most likely *Craspedolcus montezuma* belongs to the latter genus and is here excluded from *Craspedolcus*. Based on the original descriptions of the remaining species three are synonyms: *Craspedolcus obscuriventris* Enderlein, 1920 (syn. n.) of *Craspedolcus vagatus* (Smith, 1858), and *Ipobracon maculicosta* Enderlein, 1920 (syn. n.) and *Iphiaulax bhotanensis* Cameron, 1907 (syn. n.) of *Bracon simlaensis* Cameron, 1899. *Iphiaulax bhotanensis* was already informally synonymised with *Bracon simlaensis* by Yu et al. (2016). It results in 15 valid species of *Craspedolcus* s. lat. with a general distribution from Myanmar, Bhutan and India, Sundanese islands, Sulawesi up to the Philippines. So far *Craspedolcus* s. lat. is unknown from China, but among the Braconinae in the collection of the Institute of Zoology of the Chinese Academy of Sciences (Beijing) five species were found originating from southern China. The variation in the genus as defined by Quicke (1987) is extreme and the genus is likely polyphyletic after the recognition of *Serraulax* Quicke, 1987, as a separate genus. The latter genus is more similar to one part of the genus than to the other. Two very similar, but well separable genera are present in China: *Craspedolcus* s. str. and a new genus, *Maculibracon* gen. n. The latter genus was also found by the second author among material from Vietnam and Thailand deposited in the collection of Naturalis Biodiversity Center (Leiden). The biology of the new species is unknown, but members of the related genus *Campyloneurus* Szépligeti, 1900, are koinobiont endoparasitoids of larvae of Cerambycidae and Pyralidae. For the recognition of the subfamily Braconinae, see van Achterberg (1990, 1993) and for the terminology used in this paper, see van Achterberg (1988). For additional references see Yu et al. (2016).

## Materials and methods

**Figures 1–13.**
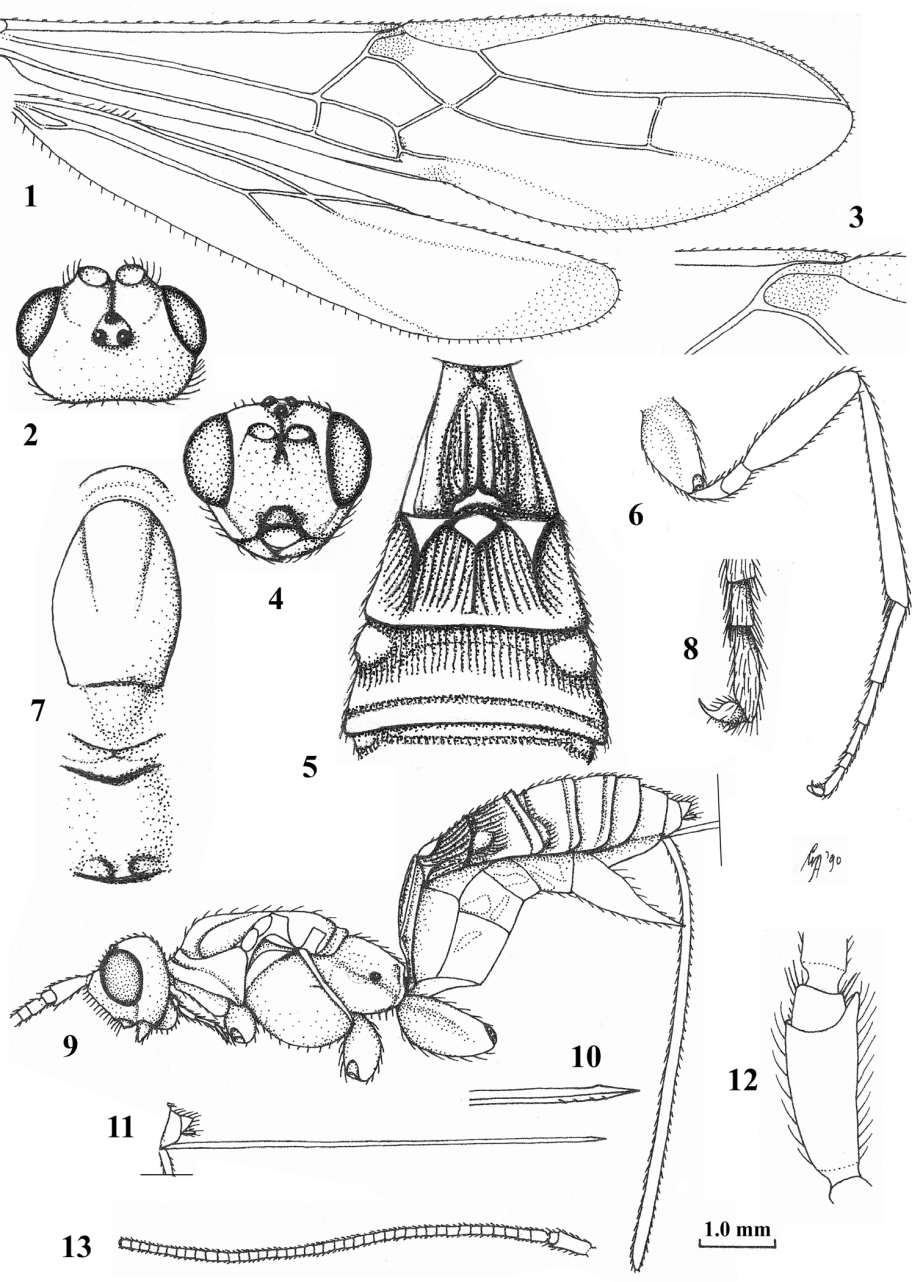
*Craspedolcus trisulcatus* Enderlein, ♀, lectotype. **1** wings **2** head dorsal **3** detail of vein 1-SR of fore wing **4** head anterior **5** first–third metasomal tergites dorsal **6** hind leg lateral **7** mesosoma dorsal **8**, outer hind claw lateral **9** habitus lateral **10** apex of ovipositor lateral **11** ovipositor **12** scapus outer side lateral **13** antenna lateral. **1, 6, 9, 11, 13**: scale-line (= 1 ×); **2–5, 7**: 2 ×; **8, 10, 12**: 5 ×.

The terminology and measurements used follow van Achterberg (1988, 1993). The following abbreviations are used: POL = postocellar line; OOL = ocular-ocellar line; OD = maximum diameter of lateral ocellus. The stigmal spot of the fore wing is the dark spot below the parastigma (Figs 1, 15). The medial length of the third metasomal tergite is measured from the posterior border of the second suture to the posterior margin of the tergite.

Photographs were made with a Keyence VHX-2000 digital microscope and the photos were slightly processed (mainly cropped and the background modified) in Photoshop CC. For the descriptions and measurements a Leica M125 stereomicroscope was used. The specimens are deposited in the Institute of Zoology, Chinese Academy of Sciences, Beijing (IZCAS) and in Naturalis Biodiversity Center, Leiden (RMNH). An asterisk indicates a new record for the country.

## Results

### Key to *Craspedolcus* auctt. in China, Vietnam and Thailand

**Table:** 

1	Scapus elongate, 2.6–2.9 times longer ventrally than its maximum width (Figs 12, 26, 41); third and fourth tergites with transverse subposterior groove (Figs 5, 9); median carina of first tergite low (Figs 5, 9); antero-lateral areas of second tergite large and touching large medio-basal area (Figs 5, 19, 33); surroundings of vein cu-a of hind wing setose; vein cu-a of fore wing subinterstitial (Fig. 1) or shortly postfurcal and perpendicular (Fig. 15); median carina of second tergite shorter and weak (Fig. 5); propodeum flat posteriorly in lateral view (Figs 9, 25, 40); vein 1r-m of hind wing shorter than vein SC+R1 (Fig. 1); antero-lateral grooves of third tergite medium-sized and remaining far removed from each other (Figs 5, 19, 33); hypopygium long and acute apically, reaching level of apex of metasoma (Figs 9, 14); second metasomal tergite below basal smooth areas densely striate (Figs 19, 33); medial area of first tergite gradually lowered anteriorly (Figs 9, 25, 40); *Craspedolcus* Enderlein, 1920 s. str.	**2**
–	Scapus stout, 1.5–2.2 times longer than its maximum width (Figs 49, 66, 74, 91); third and fourth tergites without transverse subposterior groove (Figs 47, 60, 72, 85); median carina of first metasomal tergite high anteriorly (Figs 55, 60); antero-lateral areas of second tergite minute and remaining from small medio-basal area (Figs 47, 60, 72, 85); surroundings of vein cu-a of hind wing glabrous; vein cu-a of fore wing distinctly postfurcal and inclivous (Figs 43, 56, 68, 81); median carina of second tergite long and high (Figs 47, 60, 72, 85); propodeum medio-posteriorly more or less protruding in lateral view (Figs 53, 65, 78, 90); oblique antero-lateral grooves of third tergite long and almost meeting submedially (Figs 47, 60, 72, 85); hypopygium medium-sized and subtruncate apically, not reaching level of apex of metasoma (Figs 55, 67); second metasomal tergite below basal smooth areas smooth (Figs 47, 60, 72, 85); medial area of first tergite steep anteriorly (Figs 53, 65, 78, 90)...*Maculibracon* gen. n	**3**
2	Anterior half of second metasomal tergite more or less longitudinally striate behind smooth basal areas (Fig. 19); stigmal spot medium-sized, not or hardly intruding into first discal cell of fore wing (Fig. 15); Sumatra, *China (Yunnan)	***Craspedolcus fraternus*** Enderlein, 1920
–	Anterior half of second metasomal tergite smooth, at most with some short striae near basal areas (Fig. 33); stigmal spot large, intruding into cells of fore wing below parastigma (Fig. 29); *China (Hainan)	***Craspedolcus politus* sp. n.**
3	Propodeum medio-posteriorly with smooth protuberance in lateral view (Figs 78, 90); scapus mainly yellowish brown (except for dark brown stripe on outer side) and similar to colour of head in dorsal view, rather slender and less protruding ventrally (Figs 74, 91); head less narrowed posteriorly (Figs 76, 88); medial area of first tergite low anteriorly (Figs 78, 90); slightly infuscate apical area of fore wing wide, and rather close to vein 1r-m (Figs 68, 81); stigmal spot often larger, at least up to middle of first discal cell (Figs 68, 81)	**4**
–	Propodeum medio-posteriorly with ribbed protuberance in lateral view (Figs 53, 65); scapus dark brown or blackish, darker than head in dorsal view, rather stout and more protruding ventrally (Figs 49, 66); head more narrowed posteriorly (Figs 51, 63); medial area of first tergite high anteriorly (Figs 53, 65); slightly infuscate apical area of fore wing narrow to medium-sized, remaining far from vein 1r-m (Figs 43, 56); stigmal spot smaller, up to dorsal third or half of first discal cell (Figs 43, 56)	**5**
4	Stigmal spot of fore wing larger and up to vein m-cu, enclosing nearly entire vein 1-SR+M (Fig. 81); pterostigma narrowly blackish apically (Fig. 81); medio-basal area of second tergite distinctly triangular (Fig. 85); vein 1-SR+M of fore wing dark brown (Fig. 81); Indonesia (Java), India, Bhutan, Myanmar, *Vietnam, *China (Hainan)	***Maculibracon simlaensis*** (Cameron, 1899), **comb. n.**
–	Stigmal spot of fore wing up to anterior half of first discal cell, enclosing 0.6 of vein 1-SR+M (Fig. 68); pterostigma yellow apically (Fig. 68); medio-basal area of second tergite nearly rhombic (Fig. 72); vein 1-SR+M of fore wing yellow (Fig. 68); *China (Yunnan)	***Maculibracon luteonervis* sp. n.**
5	Stigmal spot of fore wing smaller, up to anterior third of first discal cell (Fig. 56); pterostigma anteriorly dark brown (Fig. 56); medial area of first tergite gradually lowered anteriorly (Fig. 65); distal rim of fore wing distinctly infuscate and area wider (Fig. 56); medio-posterior protuberance of propodeum with two medium-sized round tubercles (Fig. 65); *China (Yunnan)	***Maculibracon hei* sp. n.**
–	Stigmal spot of fore wing larger, up to middle of first discal cell (Fig. 43); pterostigma anteriorly yellow (Fig. 43); medial area of first tergite steep anteriorly (Fig. 53); distal rim of fore wing only posteriorly narrowly infuscate (Fig. 43); medio-posterior protuberance of propodeum with small round tubercle anteriorly followed by two short transverse crests (Fig. 53); *Thailand	***Maculibracon abruptus* sp. n.**

### Descriptions

#### Craspedolcus

Taxon classificationAnimaliaHymenopteraBraconidae

Enderlein, 1920 s. str.

Figs 1–13 , 14 , 15–27 , 28 , 29–41

Craspedolcus Enderlein, 1920: 92; Shenefelt 1978: 1673; Quicke 1985: 354–357 (group A), 1987: 108; Quicke and van Achterberg 1990: 252. Type species (by original designation): Craspedolcus trisulcatus Enderlein, 1920.

##### Diagnosis.

**Figure 14.**
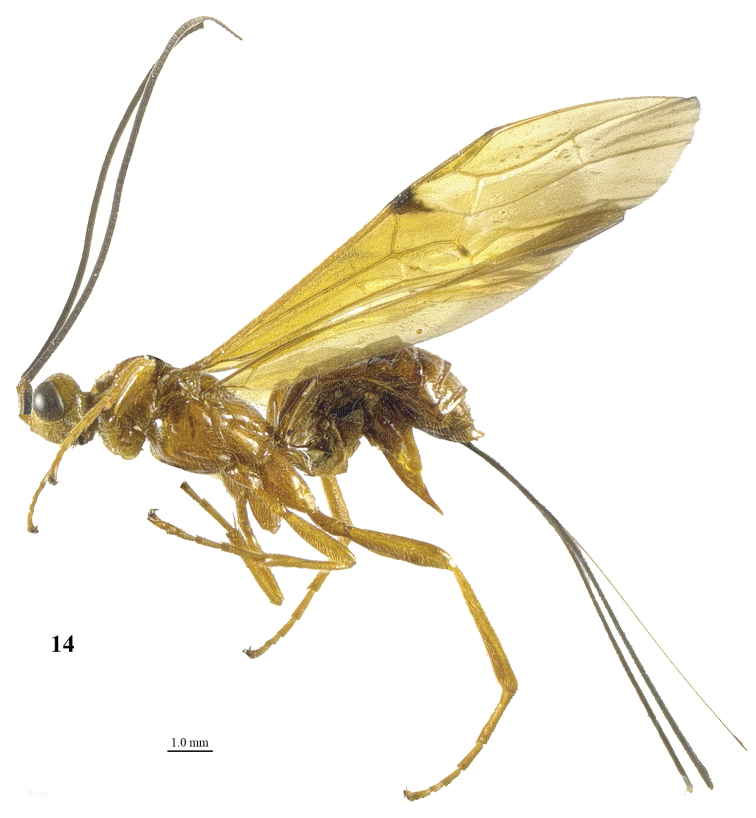
*Craspedolcus fraternus* Enderlein, ♀, China (Yunnan), habitus lateral.

**Figures 15–27.**
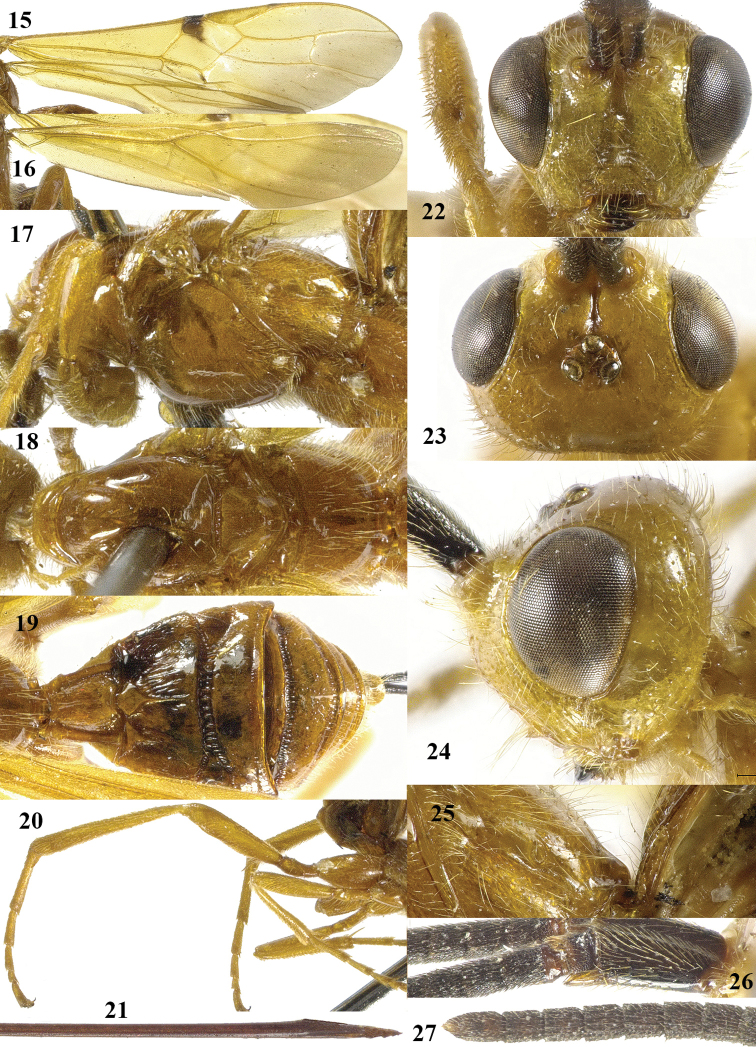
*Craspedolcus fraternus* Enderlein, ♀, China (Yunnan). **15** fore wing **16** hind wing **17** mesosoma lateral **18** mesosoma dorsal **19** metasoma dorsal **20** hind leg lateral **21** apex of ovipositor lateral **22** head, anterior **23** head, dorsal **24** head lateral **25** propodeum lateral **26** scapus outer side lateral **27** apex of antenna.

**Figure 28.**
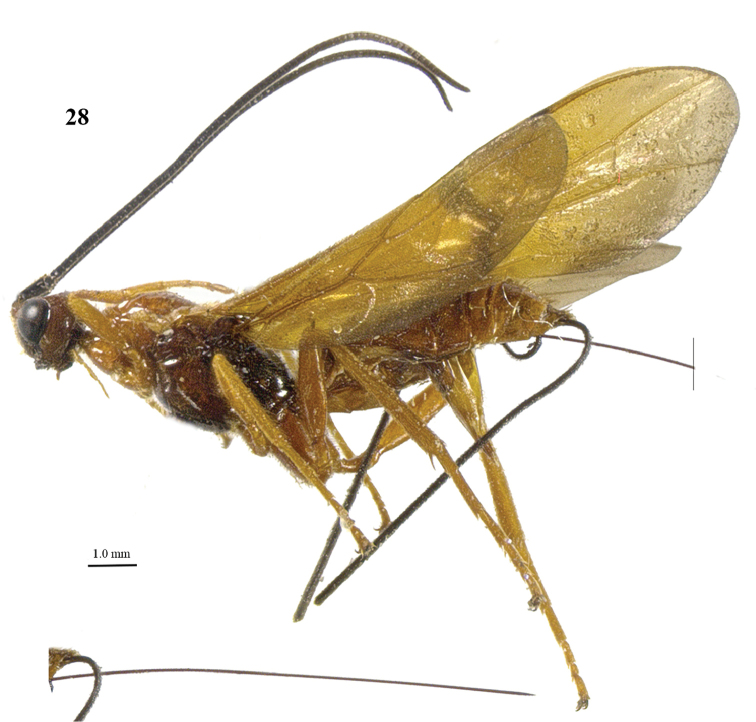
*Craspedolcus politus* sp. n., ♀, holotype, habitus lateral.

**Figures 29–41.**
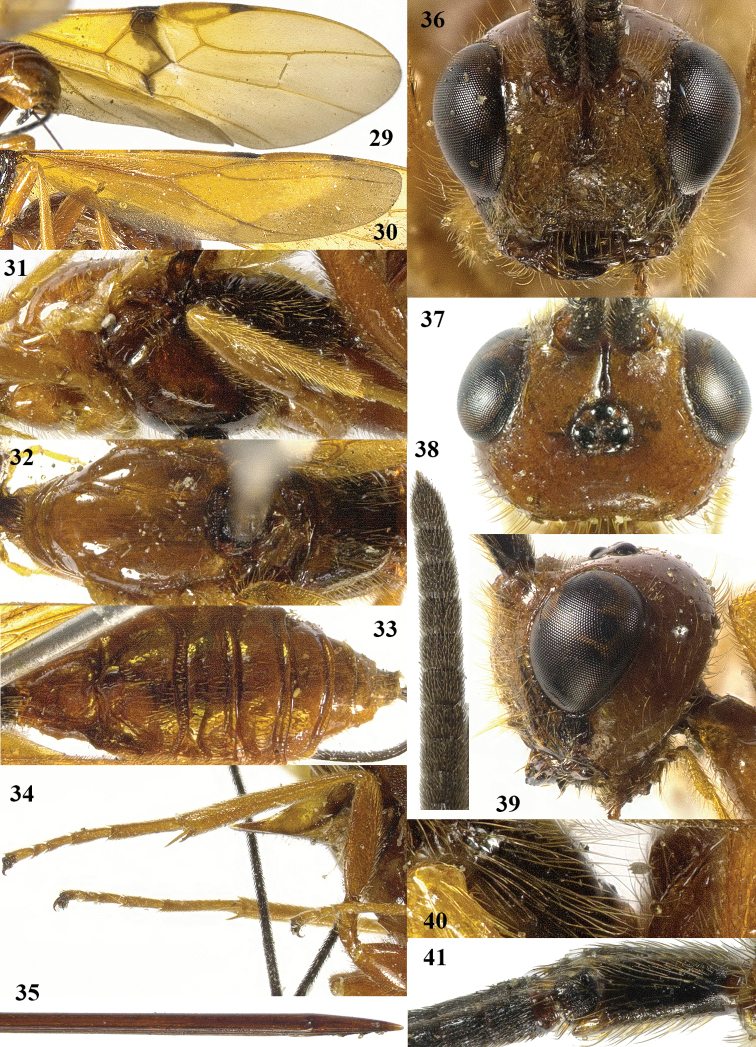
*Craspedolcus politus* sp. n., ♀, holotype. **29** fore wing **30** hind wing **31** mesosoma lateral **32** mesosoma dorsal **33** metasoma dorsal **34** hind leg lateral **35** apex of ovipositor lateral **36** head anterior **37** head dorsal **38** apex of antenna **39** head dorsal **40** propodeum lateral **41** scapus outer side lateral.

Scapus elongate, 2.6–2.9 times longer ventrally than its maximum width and protruding ventrally, rounded subbasally (Figs 12, 26, 41) and inner side without distinct ledge apically; face evenly convex; propodeum flat medio-posteriorly in lateral view (Figs 9, 25, 40); vein 3-SR of fore wing 2.5–3.4 times vein 2-SR (Figs 1, 15, 29); vein 1r-m of hind wing shorter than vein SC+R1 (Figs 1, 16, 30); vein cu-a of fore wing subinterstitial (Figs 1, 29) or shortly postfurcal and perpendicular (Fig. 15); fore wing elongate (Figs 1, 15, 29); hind wing with 3–5 subbasal bristles; surroundings of vein cu-a of hind wing setose; median carina of first tergite low and medial area gradually lowered anteriorly in lateral view (Figs 5, 9, 25, 40); second metasomal tergite below basal smooth areas striate; antero-lateral areas of second tergite large and touching large medio-basal area (Figs 5, 19, 33); median carina of second tergite medium-sized and weak (Figs 5, 19, 33); antero-lateral grooves of third tergite medium-sized and remaining far removed from each other (Figs 5, 19, 33); maximum width of third tergite 2.4–4.1 times its medial length (Figs 5, 19, 33); third and fourth tergites with transverse subposterior groove (Figs 5, 9, often crenulate but smooth in Chinese spp.); fifth and sixth tergites largely exposed and flat; subapically upper valve of ovipositor with small nodus, its lower valve fully exposed and with small teeth ventrally (Figs 10, 21, 35); hypopygium long and acute apically, reaching level of apex of metasoma (Figs 9, 14, 28); ovipositor sheath with short setae and 0.7–1.0 times as long as body.

##### Distribution.

Oriental (India, *China, Philippines, Sundanese region) and Wallacea (Sulawesi).

#### Craspedolcus fraternus

Taxon classificationAnimaliaHymenopteraBraconidae

Enderlein, 1920

Figs 14 , 15–27

Craspedolcus fraternus Enderlein, 1920: 92; Shenefelt 1978: 1673; Quicke and van Achterberg 1990: 252, 256 (lectotype designation).

##### Material.

(6 ♀; IZCAS): 1 ♀, “[**China**:] Yunnan, Xishuangbanna, Meng’a, 1050–1080 m, 11.V.1958, Shuyong Wang, No. IOZ(E)1964633”; 1 ♀, “Yunnan, Xiaomengyang, 810 m, 31.III.1957, Shuyong Wang, No. IOZ(E)1964540”; 1 ♀, “Yunnan, Xishuangbanna, Mengla, 620–650 m, 15.XI.1958, Fuji Pu, No. IOZ(E)1964636”; 1 ♀, “Yunnan, Xishuangbanna, Menghai, 1200–1600 m, 16.VIII.1957, Lingchao Zang, No. IOZ(E)1964634”; 1 ♀, “Yunnan, Simao, Mt. Puwenlong, 950–1300 m, 11.V.1957, Dahua Liu, No. IOZ(E)1964544”; 1 ♀, “Yunnan, Xishuangbanna, Gannanba, 650 m, 20.III.1957, Shuyong Wang, No. IOZ(E)1964545”.

##### Diagnosis.

Body and hind leg brownish yellow; pterostigma yellow, at most apically infuscate (Fig. 15); fore wing with a distinct but small stigmal spot near parastigma, not intruding into first discal cell of fore wing (Fig. 15) or slightly so; first tergite smooth except for its median carina (Fig. 19); anterior half of second metasomal tergite longitudinally striate (except for smooth basal areas; Fig. 19) and remainder of tergite smooth; third tergite smooth basally and its transverse subposterior groove smooth (Fig. 19); length of ovipositor sheath 0.8–1.0 times both length of body and of fore wing.

*Craspedolcus fraternus* and *Craspedolcus politus* are the only species of *Craspedolcus* s. str. having the transverse subposterior groove and basal half of the third tergite smooth, the first tergite smooth, shiny and its median carina low, second tergite smooth posteriorly, and ovipositor sheath with yellowish setae. *Craspedolcus fraternus* has a smaller stigmal spot than *Craspedolcus politus* (Fig. 15 *versus* Fig. 29) and the anterior half of the second tergite more extensively striate (Fig. 19 *versus* Fig. 33).

*Variation*. Length of body of female 9.5–14.4 mm, of fore wing of female 11.0–15.2 mm, and of ovipositor sheath 9.6–15.0 mm; antenna of female with 68 (1), 69 (2), 71 (1) segments; vein 3-SR of fore wing 2.4–2.9 times vein 2-SR; length of first tergite 1.2–1.3 times its apical width; length of ovipositor sheath 0.82–0.99 times fore wing; mesosoma and metasoma ventrally yellowish brown or infuscated; fore wing with irregular stigmal spot up to vein 1-SR+M or apical 0.2 of first submarginal cell; ventrally apex of scapus more or less yellowish; vein cu-a of fore wing interstitial or narrowly postfurcal; extent of apical infuscation of hind wing as figured (Fig. 16) or somewhat less; face colour similar to that of mesoscutum or distinctly paler; vein 1-SR+M of fore wing yellow or partly brown; fore tarsus 1.3–1.4 times as long as fore tibia; long ventral setae of scapus appressed or erect.

##### Distribution.

Indonesia (Sumatra), *China (Yunnan).

#### Craspedolcus politus sp. n.

Taxon classificationAnimaliaHymenopteraBraconidae

http://zoobank.org/1D7F176D-641D-4CEB-9306-EE7CDBD67DB0

Figs 28 , 29–41

##### Type material.

Holotype, ♀ (IZCAS), “[**China**:] Hainan, Jianfengling, 4.V.1985, Maobin Gu, No. IOZ(E)1964586”. Paratypes (3 ♀; IZCAS): 1 ♀, same data as holotype, but No. IOZ(E)1964591; 1 ♀ id., 4.IV.1984, Youdong Lin, No. IOZ(E)1964590; 1 ♀, Hainan, [locality unknown], 8.VII.1982, Youdong Lin, No. IOZ(E)1964610.

##### Diagnosis.

Body and hind leg yellowish brown; pterostigma yellow, but apically dark brown (Fig. 29); fore wing with stigmal spot up to vein CU1b, intruding in cells of fore wing below parastigma and included veins dark brown (Fig. 29); first tergite smooth except for its median carina and few striae (Fig. 33); second metasomal tergite smooth except for crenulae or short striae near outer side of antero-lateral areas and below nearly rhombical medio-basal area (Fig. 33); third tergite (including its transverse subposterior groove and both antero-lateral grooves) smooth (Fig. 33); length of ovipositor sheath 0.9 times body. For the separation from other species of *Craspedolcus*, see the diagnosis of *Craspedolcus fraternus* Enderlein.

##### Description.

Holotype, ♀, length of body 12.0 mm, of fore wing 12.8 mm, of ovipositor sheath 10.3 mm.

*Head*. Antenna 0.85 times as long as fore wing, with 71 segments; apical antennal segment with short spine, scapus slender, parallel-sided and distinctly protruding ventro-apically, with narrow indistinct apical ledge at inner side and basally gradually narrowed, its ventral setae erect (Figs 38, 41); third, fourth and penultimate segments 1.8, 1.2 and 1.1 times their maximum width, respectively; length of maxillary palp 0.8 times height of head; eye not emarginated (Fig. 36); face weakly and evenly convex, remotely finely punctate and with long erect yellowish setae; clypeus flat, superficially rugose, dorsally with weak carina and ventral margin thin and lamelliform protruding, with a row of long yellowish setae ventrally; hypoclypeal depression 0.5 times as wide as minimum width of face (Fig. 36); frons shallowly concave behind antennal sockets, with deep median groove, smooth except for a few punctures laterally (Fig. 36); vertex smooth except for few punctures and weakly convex, glabrous; OOL:diameter of posterior ocellus:POL = 30:7:5; in dorsal view length of eye 1.4 times temple; temples subparallel-sided behind eyes, with spaced setiferous punctures and long setae (Figs 37, 39); malar suture absent present and curved; length of malar space 0.8 times basal width of mandible; mandible twisted and with two wide teeth.

*Mesosoma*. Length of mesosoma 1.9 times its height (Fig. 31); side of pronotum shiny and smooth; propleuron with spaced punctures; pronotum vertical anteriorly and with a shallow groove and no antescutal depression; mesopleuron smooth and glabrous, anteriorly punctulate and sparsely setose; mesosternal sulcus smooth and narrow; metapleuron smooth and with long setae, convex; mesoscutum glabrous except some setae near notaulic courses, shiny and smooth; notauli shallowly impressed, smooth; scutellar sulcus present and with distinct fine crenulae; scutellum nearly flat anteriorly and smooth; side of scutellum smooth; metanotum medio-anteriorly with short carina, posteriorly evenly convex and smooth; propodeum smooth, with many long setae and evenly convex, medio-apically smooth in lateral view (Fig. 40).

*Wings*. Fore wing (Fig. 29): m-cu 0.8 times as long as 1-M; 1-SR+M sharply angled after arising from 1-M, 1.5 times as long as 1-M; 3-SR weakly curved, and SR1 straight; r:3-SR:SR1 = 6:36:41; 2-SR:3-SR:r-m = 13:35:13; r-m largely sclerotised; 1-CU1 widened and 0.06 times 2-CU1; cu-a vertical; CU1b narrower than 3-CU1. Hind wing (Fig. 30): with 4 coarse subbasal bristles on C+SC+R and with 3 hamuli on R1; SR weakly curved basally and marginal cell parallel-sided apically; subbasal cell near cu-a setose; 1r-m straight and 0.9 times as long as SC+R1; 2-SC+R 1.3 times longer than wide.

*Legs*. Tarsal claws simple and with long bristly setae ventrally; fore tarsus 1.5 times as long as fore tibia and tibia bristly setose and pimply anteriorly; length of femur, tibia and basitarsus of hind leg 4.2, 10.1 and 6.2 times their maximum width, respectively; hind tibia with dense appressed setae (Fig. 34); hind tibial spurs 0.3 and 0.4 times as long as hind basitarsus; inner side of hind tibia and tarsus densely bristly setose.

*Metasoma*. Length of first tergite 1.2 times its apical width, medial area low anteriorly, dorso-lateral carinae strongly developed, medial area smooth except for low median carina and few striae; second tergite largely smooth (including deep oblique anterior grooves) except for median carina connected to nearly rhombical medio-basal area and weak crenulae near medio-basal area and outer side of antero-lateral triangular areas (Fig. 33); second metasomal suture strongly crenulate, laterally narrowed and oblique; medially second tergite about as long as third tergite; maximum width of third tergite 3.5 times its medial length; third–fifth tergites smooth and with smooth transverse subposterior groove and antero-lateral grooves; ovipositor sheath 0.80 times as long as fore wing and 0.9 times body; hypopygium just surpassing apex of metasoma.

*Colour*. Yellowish brown; antenna (including entire scapus), mandible apically, stemmaticum, and ovipositor sheath dark brown or black; posterior half of mesosoma largely infuscate; apical 0.2 of pterostigma dark brown; remainder of pterostigma and wing membrane yellow, but fore wing with irregular stigmal spot up to vein CU1b, including dark brown veins 1-SR, 1-SR+M, m-cu and 3-CU1 and apically wings with wide infuscate area; remainder of veins brownish yellow (Figs 29, 30).

*Variation*. Length of body of female 10.4–12.0 mm, of fore wing of female 12.0–13.4 mm, and of ovipositor sheath 9.3–12.0 mm; antenna of female with 71 (2), 68 (1) segments; vein 3-SR of fore wing 2.6–3.0 times vein 2-SR; length of first tergite 1.2–1.3 times its apical width; length of ovipositor sheath 0.78–0.90 times fore wing; mesosoma and metasoma ventrally yellowish brown or infuscated; infuscate apical part of fore wing up to vein r-m or somewhat narrower; ventrally apex of scapus more or less yellowish vein cu-a of fore wing interstitial or narrowly postfurcal; fore tarsus 1.4–1.5 times as long as fore tibia; apical infuscation of hind wing as figured (Fig. 30) or somewhat wider; face colour similar to that of mesoscutum or paler.

##### Distribution.

China (Hainan).

##### Etymology.

Named “politus” (Latin for “made smooth”) because of the smooth transverse subposterior grooves of the metasoma and the smooth third tergite.

#### Maculibracon gen. n.

Taxon classificationAnimaliaHymenopteraBraconidae

http://zoobank.org/7FFC7170-6E03-45F7-9177-71052DFD6FEA

Figs 42 , 43–54 , 55 , 56–66 , 67 , 68–79 , 80 , 81–91

Craspedolcus Enderlein, 1920: 92 (p.p.); Shenefelt 1978: 1673 (p.p.); Quicke 1985: 354–357 (group B), 1987: 108 (p.p.); Quicke and van Achterberg 1990: 252 (p.p.).

##### Type species.

*Maculibracon abruptus* sp. n.

##### Diagnosis.

**Figure 42.**
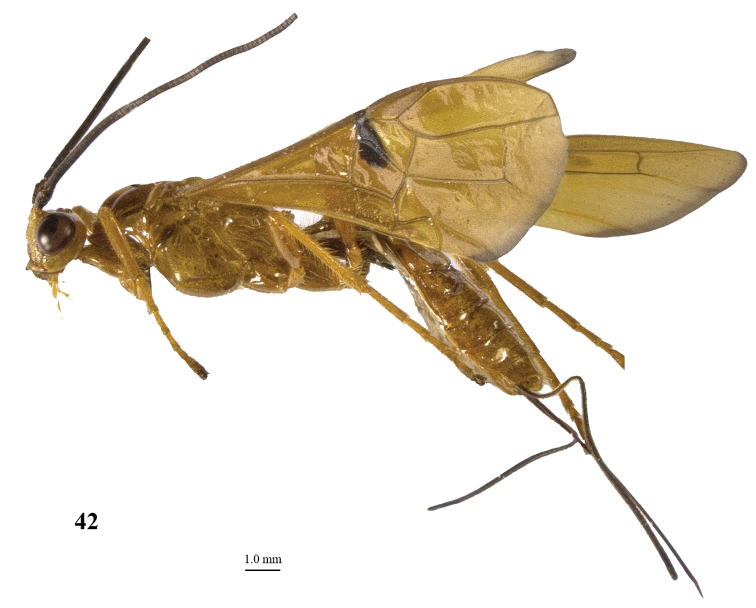
*Maculibracon abruptus* sp. n., ♀, holotype, habitus lateral.

**Figures 43–54.**
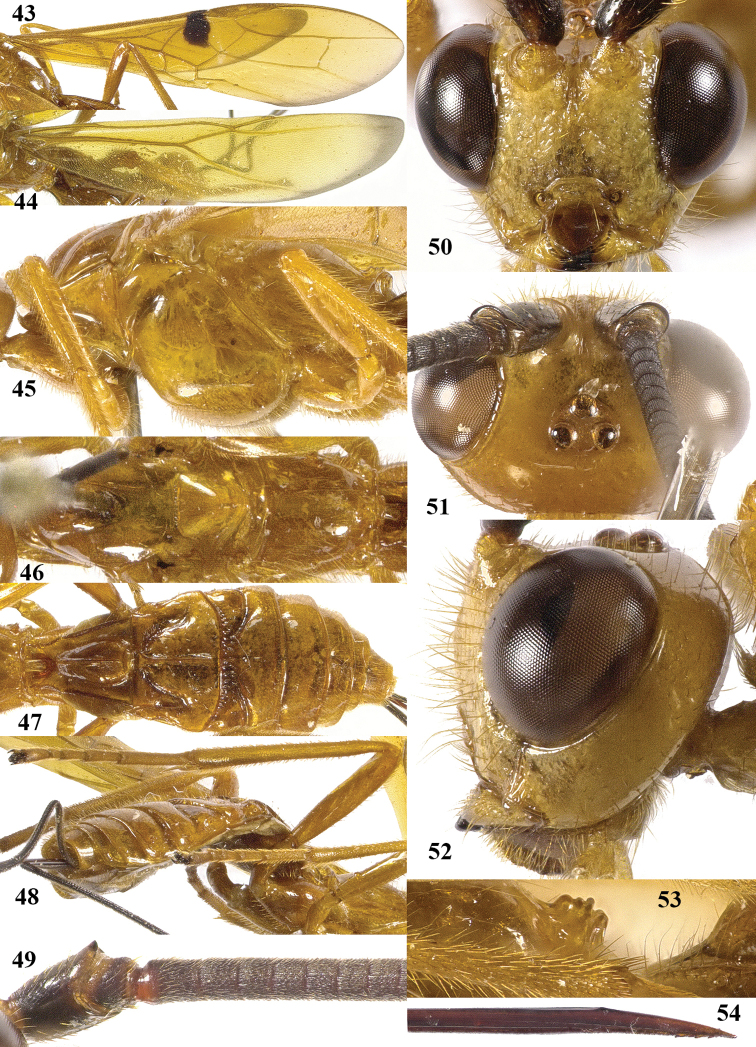
*Maculibracon abruptus* sp. n., ♀, holotype. **43** fore wing **44** hind wing **45** mesosoma lateral **46** mesosoma dorsal **47** metasoma dorsal **48** hind leg lateral **49** scapus outer side lateral **50** head anterior **51** head dorsal **52** head lateral **53** propodeum lateral **54** apex of ovipositor lateral.

**Figure 55.**
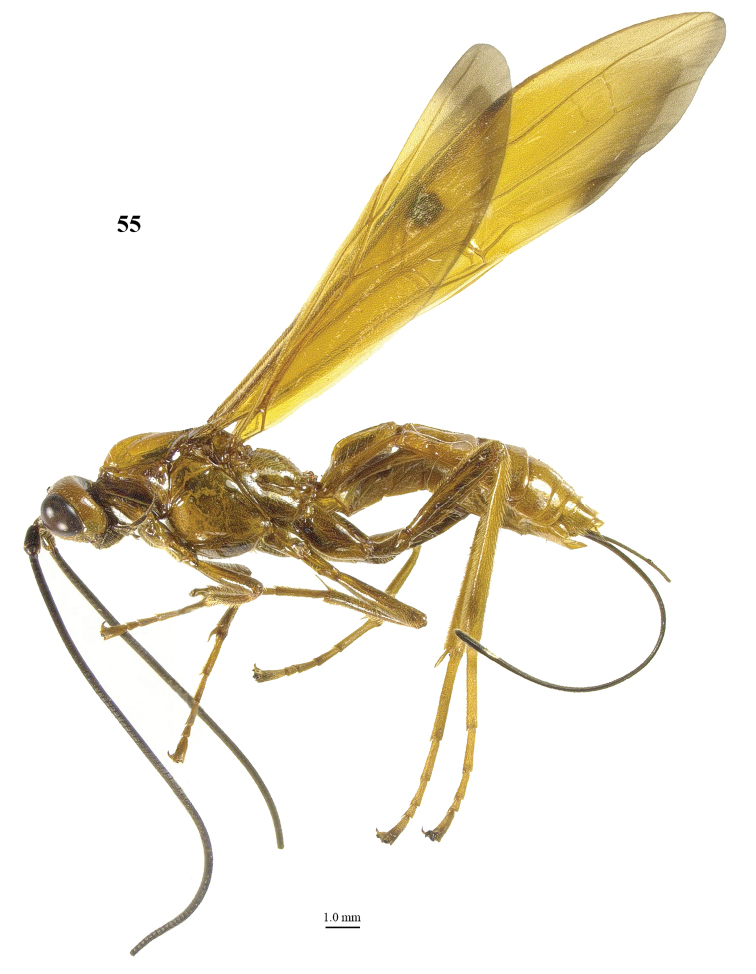
*Maculibracon hei* sp. n., ♀, holotype, habitus lateral.

**Figures 56–66.**
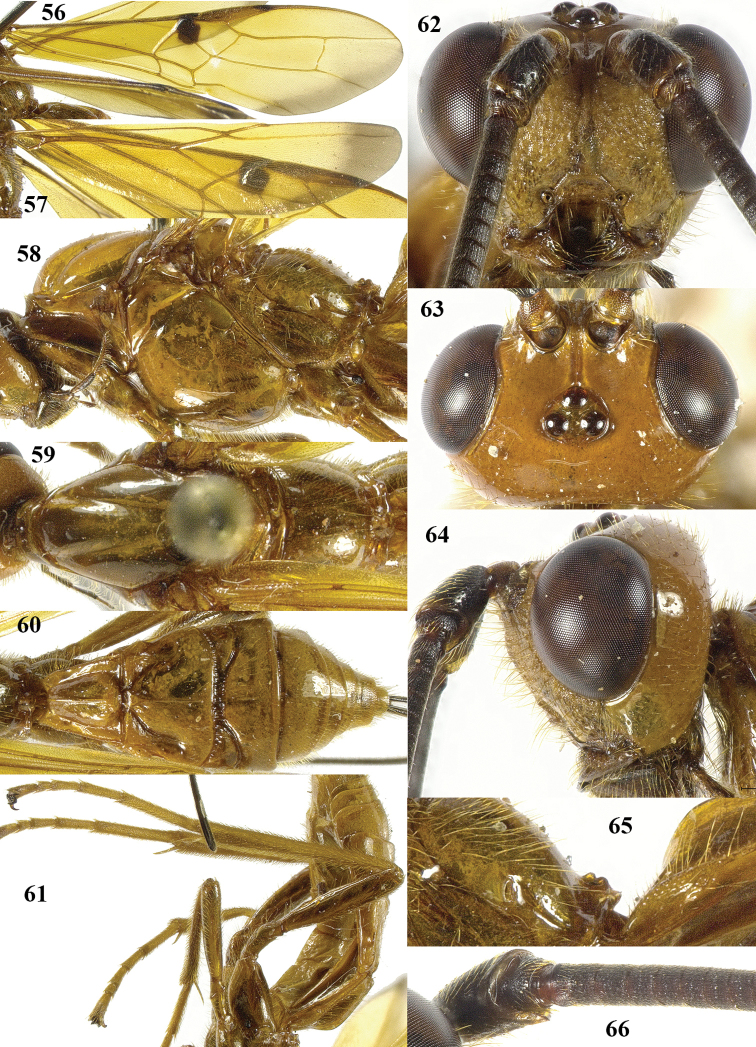
*Maculibracon hei* sp. n., ♀, holotype. **56** fore wing **57** hind wing **58** mesosoma lateral **59** mesosoma dorsal **60** metasoma dorsal **61** hind leg lateral **62** head anterior **63** head dorsal **64** head lateral **65** propodeum lateral **66** scapus outer side lateral.

**Figure 67.**
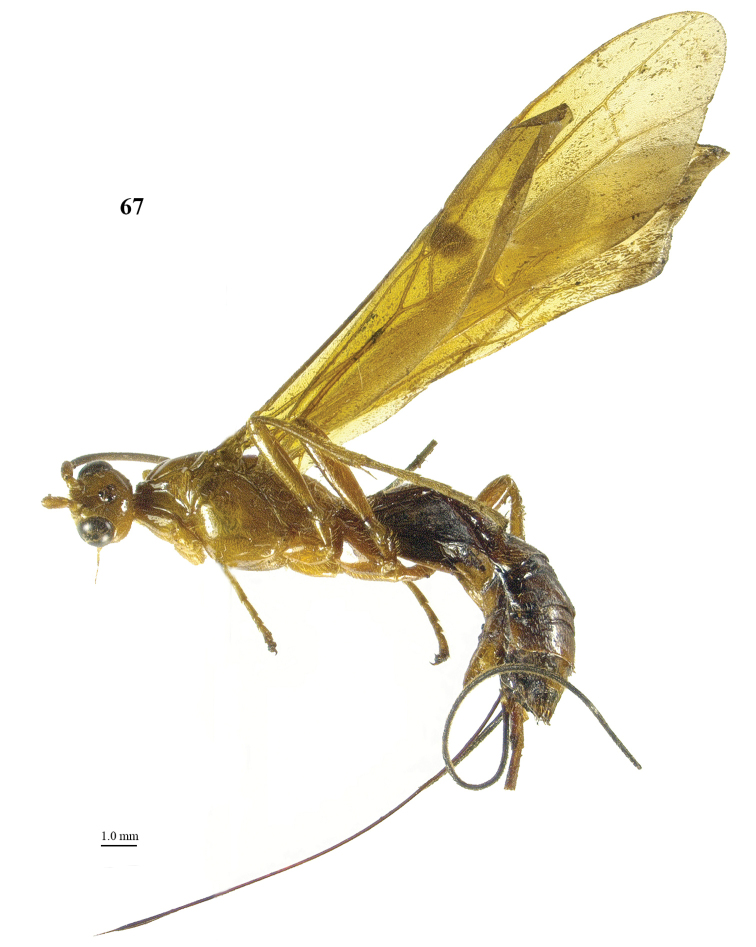
*Maculibracon luteonervis* sp. n., ♀, holotype, habitus lateral.

**Figures 68–79.**
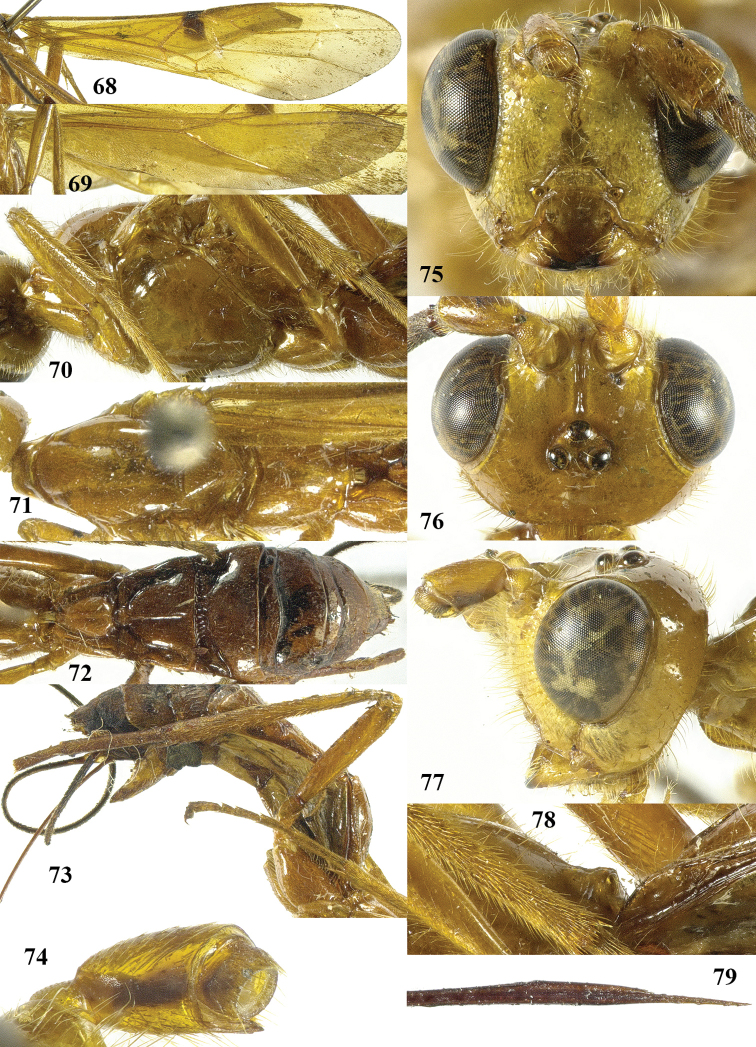
*Maculibracon luteonervis* sp. n., ♀, holotype. **68** fore wing **69** hind wing **70** mesosoma lateral **71** mesosoma dorsal **72** metasoma dorsal **73** hind leg lateral **74** scapus outer side lateral **75** head anterior **76** head dorsal **77** head lateral **78** propodeum lateral **79** apex of ovipositor lateral.

**Figure 80.**
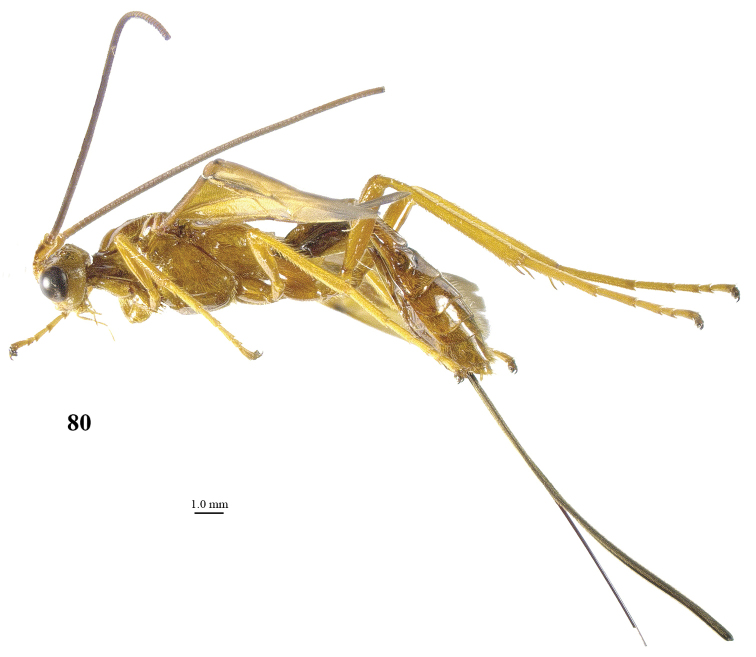
*Maculibracon simlaensis* (Cameron), ♀, Vietnam, habitus lateral.

**Figures 81–91.**
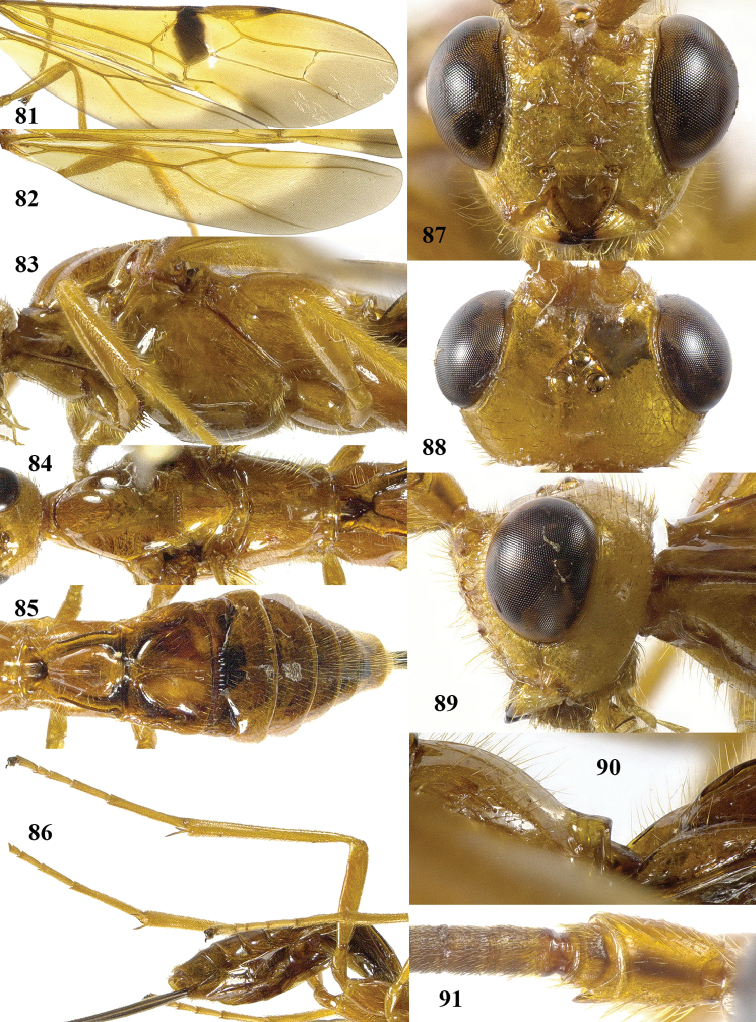
*Maculibracon simlaensis* (Cameron), ♀, Vietnam. **81** fore wing **82** hind wing **83** mesosoma lateral **84** mesosoma dorsal **85** metasoma dorsal **86** hind leg lateral **87** head anterior **88** head dorsal **89** head lateral **90** propodeum lateral **91** scapus outer side lateral.

Scapus stout, 1.5–2.2 times longer than its maximum width and protruding ventrally (Figs 49, 66, 74, 91), rounded subbasally and inner side at most with narrow ledge apically; face flattened medially; propodeum medio-posteriorly more or less protruding in lateral view (Figs 53, 65, 78, 90); vein 3-SR of fore wing 2.4–2.8 times vein 2-SR (Figs 43, 56, 68, 81); vein 1r-m of hind wing 1.0–1.6 times as long as vein SC+R1 (Figs 44, 57, 69, 82); vein 3-SR of fore wing 0.9 times as long as vein SR1 or longer (Figs 43, 56, 68, 81); hind wing with 4-5 subbasal bristles; vein cu-a of fore wing strongly postfurcal and inclivous (Figs 43, 56, 68, 81); fore wing elongate (Figs 43, 56, 68, 81); median carina of first tergite high and medial area steep anteriorly in lateral view (Figs 53, 65, 78, 90); second metasomal tergite below basal areas smooth; antero-lateral areas of second tergite minute and remaining from small medio-basal area (Figs 47, 60, 72, 85); median carina of second tergite long and high (Figs 47, 60, 72, 85); strongly oblique antero-lateral grooves of third tergite long and almost meeting submedially (Figs 47, 60, 72, 85); maximum width of third tergite 2.7–3.2 times its medial length (Figs 47, 60, 72, 85); third and fourth tergites without transverse subposterior groove (Figs 47, 60, 72, 85); fifth and sixth tergites largely exposed and flat; subapically upper valve of ovipositor with small nodus, its lower valve fully exposed and with small teeth ventrally (Figs 54, 79); hypopygium medium-sized and subtruncate apically, not reaching level of apex of metasoma (Figs 42, 55, 67, 80); ovipositor sheath narrow, with short yellowish setae and 0.4–0.8 times as long as body.

##### Distribution.

Oriental (India, Bhutan, Myanmar, *Thailand, *Vietnam, *China, Philippines, Sundanese region).

##### Etymology.

Name derived from “macula” (Latin for “spot, mark”) and the generic name *Bracon*, because of the conspicuous dark spot of the fore wing. Gender: masculine.

##### Notes.

Quicke (1985) already indicated that *Craspedolcus* was heterogeneous; he divided the genus in two groups. Group A includes the type species (= *Craspedolcus* s. str.) and group B is described in this paper as new genus. The new genus is similar to the Afrotropical genus *Serraulax* Quicke, 1987, but differs by having the inner apex of scapus simple or with a minor ledge (*versus* with moderately wide ledge formed by a false margin in *Serraulax*), vein 1r-m of hind wing about as long as vein SC+R1 (*versus* distinctly longer), vein 2-SC+R of hind wing hardly longer than wide (*versus* distinctly longer than wide), vein cu-a of fore wing distinctly inclivous (*versus* more or less perpendicular), median carina of first tergite present anteriorly (*versus* absent anteriorly), second tergite smooth (*versus* distinctly longitudinally striate) and third tergite without subposterior transverse groove (*versus* with subposterior groove present).

Some species of the genus *Hybogaster* Szépligeti, 1906, are very similar to the new genus (e.g. first tergite with strong median carina and medial area protuberant anteriorly, wings elongate and mainly yellow, and fore wing with a dark stigmal spot). They differ by having the second tergite spaced longitudinally striate, the scapus short ovoid and not protruding ventrally, antero-lateral grooves of third tergite subvertical and indistinct because of the depression near it, vein 1-SR+M of fore wing straight or nearly so and upper valve of ovipositor without subapical nodus, depressed and covering the narrow and ventrally smooth lower valve. The genus *Hybogaster* remains unknown from China; the holotype of the only reported species, *Hybogaster zebripterae* Wang & Chen, 2008, from China (Fujian) has been examined and proved to belong to *Iphiaulax* Foerster, 1863, comb. n.

The following names form new combinations in *Maculibracon* gen. n.: *Bracon lepcha* Cameron, 1899; *Bracon phaedo* Cameron, 1899; *Bracon simlaensis* Cameron, 1899; *Iphiaulax bhotanensis* Cameron, 1907; *Iphiaulax laertius* Cameron, 1903; *Iphiaulax leptopterus* Cameron, 1903; *Iphiaulax lineaticarinatus* Cameron, 1907; *Ipobracon lissotomus* Roman, 1914; *Ipobracon maculicosta* Enderlein, 1920, and *Ipobracon pallidicornis* Roman, 1914.

#### Maculibracon abruptus sp. n.

Taxon classificationAnimaliaHymenopteraBraconidae

http://zoobank.org/90A521BE-A3BD-4B2C-9576-C7C8CF0C9AF6

Figs 42 , 43–54

##### Type material.

Holotype, ♀ (RMNH), “Peninsular **Thailand**, NW [of] Phuket, Nai Yang, 26.II-4.III.[20]07, [S.] Risch”.

##### Diagnosis.

Entire scapus dark brown, rather stout and rather protruding ventrally; head distinctly narrowed posteriorly (Fig. 51); pterostigma entirely yellow; stigmal spot of fore wing rather large, up to middle of first discal cell (Fig. 43); wing membrane of fore wing yellow distally only posteriorly narrowly infuscate remaining far from vein 1r-m (Fig. 43); medio-posterior protuberance of propodeum with small round tubercle anteriorly followed by two short transverse crests, ribbed in lateral view (Fig. 53); medial area of first tergite high and steep anteriorly (Fig. 53); body and hind leg brownish yellow; length of ovipositor sheath 0.5 times fore wing and 0.6 times body. Similar to *Maculibracon leptopterus* (Cameron, 1903) because of dark scapus, stigmal spot up to middle of first discal cell and anteriorly steep medial area of first tergite. The new species differs by the longer ovipositor sheath (0.7 times *versus* 0.4 times in *Maculibracon leptopterus* according to the original description), the sculpture of the second metasomal tergite (absent *versus* crenulate or striate near smooth antero-lateral areas), colour of the setae of the face (yellow *versus* fuscous), the shape of the medio-posterior protuberance of the propodeum (posteriorly with two short transverse crests *versus* no crests) and the colour of the pterostigma (yellow apically *versus* dark brown).

##### Description.

Holotype, ♀, length of body 14.5 mm, of fore wing 16.2 mm, of ovipositor sheath 8.7 mm.

*Head*. Antenna incomplete, with 60 segments remaining; scapus rather stout, 1.6 times longer than wide and distinctly emarginated apically, slightly longer ventrally than dorsally in lateral view, with a narrow apical ledge at inner side and gradually narrowed basally (Fig. 49); third and fourth segments 1.5 and 0.9 times their maximum width, respectively; length of maxillary palp as long as the height of head; inner side of eye not emarginated but slightly sinuate (Fig. 50); face moderately convex but flattened medially and medio-dorsally with weak median crest, sparsely punctate, and with long erect setae; clypeus flat, nearly smooth, dorsally with coarse curved carina and ventral margin thin and lamelliform, with few long setae ventrally; hypoclypeal depression 0.4 times as wide as minimum width of face (Fig. 50); frons shallowly concave behind antennal sockets, smooth, except for a rather shallow median groove (Fig. 50); vertex smooth, except for with some shallow setiferous punctures with long setae and shiny; stemmaticum distinctly protruding; OOL:diameter of posterior ocellus:POL = 31:15:9; in dorsal view length of eye 2.1 times temple; temples linearly narrowed behind eyes, with some long setae, punctate dorsally and sparsely punctate ventrally (Fig. 51, 52); malar suture shallow, curved; length of malar space 0.8 times basal width of mandible; mandible twisted, both teeth robust and subequal.

*Mesosoma*. Length of mesosoma 1.7 times its height; side of pronotum shiny and smooth (Fig. 45); propleuron with spaced punctures; pronotum vertical anteriorly, with a shallow pronope and groove and narrow antescutal depression; mesopleuron smooth and glabrous, except for an oblique band with punctures and short setae; mesosternal sulcus smooth and narrow; metapleuron smooth and with long setae, convex; mesoscutum glabrous except some setae near notaulic courses, shiny and smooth; notauli smooth, only distinct anteriorly and medially shallowly impressed; scutellar sulcus present and with distinct fine crenulae; scutellum distinctly convex and smooth except for some punctures; side of scutellum smooth; metanotum medio-anteriorly with short carina, posteriorly evenly convex and smooth; propodeum distinctly remotely punctate, with long setae, evenly convex but medio-posteriorly with protuberance consisting of small round tubercle anteriorly followed by two short transverse crests (Fig. 46), ribbed in lateral view (Fig. 53).

*Wings*. Fore wing (Fig. 43): m-cu 0.8 times as long as 1-M; 1-SR+M weakly bent subbasally 1.3 times as long as 1-M; 3-SR weakly curved, and SR1 straight; r:3-SR:SR1 = 10:56:46; 2-SR:3-SR:r-m = 20:54:20; r-m largely sclerotised; 1-CU1 slightly widened and 0.14 times 2-CU1; cu-a weakly inclivous; CU1b nearly as wide as 3-CU1. Hind wing (Fig. 44): with 4 coarse subbasal bristles on C+SC+R and with 3 hamuli on R1; SR weakly curved basally and marginal cell parallel-sided apically; area near cu-a glabrous; 1r-m straight and 1.6 times as long as SC+R1; 2-SC+R as wide as long.

*Legs*. Tarsal claws simple and with long bristly setae ventrally; length of femur, tibia and basitarsus of hind leg 5.0, 12.0 and 7.3 times their maximum width, respectively; hind tibia with dense and rather appressed setae; hind tibial spurs 0.3 and 0.4 times as long as hind basitarsus; inner side of hind tibia and tarsus densely bristly setose (Fig. 48).

*Metasoma*. Length of first tergite 1.3 times its apical width, dorso-lateral carinae strongly developed, medial area smooth except for high median carina and medial area steep anteriorly (Figs 47, 53); second tergite smooth (including deep oblique anterior grooves) except strong median carina connected to minute triangular medio-basal area, antero-lateral triangular areas large (Fig. 47); second metasomal suture strongly crenulated, laterally narrowed and weakly up curved; medially second tergite 1.6 times longer than third tergite; maximum width of third tergite 3.0 times its medial length; third–fifth tergites smooth and without transverse subposterior groove and with long antero-lateral grooves; ovipositor sheath 0.54 times as long as fore wing and 0.6 times body; hypopygium ending just anterior of apex of metasoma.

*Colour*. Yellowish brown; antenna (but small part of scapus brown) and mandible apically dark brown; ovipositor sheath blackish with yellow setae; stigmal spot medium-sized, up to dorsal third of first discal cell (Fig. 43); veins yellow except dark brown basal half of 1-SR+M and most of 1-SR; wing membrane yellow, except for stigmal spot and infuscate narrow apical margin (Figs 43, 44).

##### Distribution.

*Thailand.

##### Etymology.

Named after the medio-anterior steep part of the first tergite: “abruptus” is Latin for “steep”.

#### Maculibracon hei sp. n.

Taxon classificationAnimaliaHymenopteraBraconidae

http://zoobank.org/859877FC-DF4C-4BF8-BFE5-08CE11C94144

Figs 55 , 56–66

##### Type material.

Holotype, ♀ (IZCAS), “[**China**:] Yunnan, Lancang, 1000 m, 30.VII.1957, Lingchao Zang, No. IOZ(E)1964638”.

##### Diagnosis.

Entire scapus dark brown or black, rather stout and more protruding ventrally; head rather directly narrowed posteriorly (Fig. 63); stigmal spot rather small, up to anterior third of first discal cell (Fig. 56); wing membrane yellow with slightly infuscate apical area of fore wing medium-sized, but remaining far from vein 1r-m (Figs 56, 57); pterostigma anteriorly dark brown and remainder yellow; medio-posterior protuberance of propodeum with two medium-sized round tubercles, ribbed in lateral view (Fig. 65); medial area of first tergite high anteriorly and gradually lowered basally (Figs 60, 65); body and hind leg brownish yellow; length of ovipositor sheath 0.6 times fore wing and 0.7 times body. Similar to *Maculibracon leptopterus* (Cameron, 1903) because of dark scapus and medium-sized stigmal spot. The new species differs by the longer ovipositor sheath (0.7 times *versus* 0.4 times in *Maculibracon leptopterus* according to the original description), the sculpture of the second metasomal tergite (absent *versus* crenulate or striate near smooth antero-lateral areas), colour of the setae of the face (yellow *versus* fuscous), the size of the stigmal spot (up to anterior third of first discal cell (*versus* up to middle of cell) and the colour of the pterostigma (anteriorly dark brown *versus* yellow except dark brown apex).

##### Description.

Holotype, ♀, length of body 17.2 mm, of fore wing 17.8 mm, of ovipositor sheath 11.2 mm.

*Head*. Antenna incomplete, left antenna with 83 segments remaining, right antenna with 67 segments remaining; scapus rather stout, 1.5 times longer than wide and distinctly emarginate apically, longer ventrally than dorsally in lateral view, with a narrow apical ledge at inner side and gradually narrowed basally (Fig. 66); third and fourth segments 1.7 and 1.1 times their maximum width, respectively; length of maxillary palp as long as height of head; inner side of eye not emarginated but slightly sinuate (Fig. 63); face moderately convex but flattened medially and medio-dorsally with weak median crest, coarsely and densely punctate, and with long erect setae; clypeus flat, rugose, dorsally with coarse curved carina and ventral margin thin and lamelliform, with few long setae ventrally; hypoclypeal depression 0.3 times as wide as minimum width of face (Fig. 62); frons shallowly concave behind antennal sockets, rugose, with a rather shallow median groove (Fig. 62); vertex smooth, with few long setae and shiny; stemmaticum distinctly protruding; OOL:diameter of posterior ocellus:POL = 25:10:8; in dorsal view length of eye 2.3 times temple; temples directly narrowed behind eyes, with some long setae, punctate dorsally and sparsely punctate ventrally (Fig. 63, 64); malar suture shallow, curved; length of malar space 0.8 times basal width of mandible; mandible twisted, both teeth robust and subequal.

*Mesosoma*. Length of mesosoma 1.8 times its height (Fig. 58); side of pronotum shiny and smooth; propleuron with spaced punctures; pronotum vertical anteriorly, with a shallow pronope and groove and narrow antescutal depression; mesopleuron smooth and glabrous, except for an oblique band with punctures and short setae; mesosternal sulcus smooth and narrow; metapleuron smooth and with long setae, convex; mesoscutum glabrous except some setae near notaulic courses, shiny and smooth; notauli smooth, only distinct anteriorly and medially shallowly impressed; scutellar sulcus present and with distinct fine crenulae; scutellum distinctly convex and smooth except for some very sparse punctulation; side of scutellum smooth; metanotum medio-anteriorly with short carina, posteriorly evenly convex and smooth; propodeum distinctly remotely punctate, with long setae, evenly convex but medio-posteriorly with protuberance consisting of small medium-sized round tubercles anteriorly followed by two short transverse crests (Fig. 59), ribbed in lateral view (Fig. 65).

*Wings*. Fore wing (Fig. 56): m-cu 0.6 times as long as 1-M; 1-SR+M angularly bent subbasally 1.2 times as long as 1-M; 3-SR weakly curved, and SR1 straight; r:3-SR:SR1 = 5:29:24; 2-SR:3-SR:r-m = 11:29:11; r-m largely sclerotised; 1-CU1 slightly widened and 0.17 times 2-CU1; cu-a weakly inclivous; CU1b nearly as wide as 3-CU1. Hind wing (Fig. 57): with 4 or 5 coarse subbasal bristles on C+SC+R and with 3 hamuli on R1; SR weakly curved basally and marginal cell subparallel-sided apically; area near cu-a glabrous; 1r-m straight and 1.3 times as long as SC+R1; 2-SC+R as wide as long.

*Legs*. Tarsal claws simple and with long bristly setae ventrally; length of femur, tibia and basitarsus of hind leg 5.2, 12.5 and 7.9 times their maximum width, respectively; hind tibia with dense and rather appressed setae; hind tibial spurs 0.3 and 0.4 times as long as hind basitarsus; inner side of hind tibia and tarsus densely bristly setose (Fig. 61).

*Metasoma*. Length of first tergite 1.3 times its apical width, dorso-lateral carinae strongly developed, medial area smooth except for high median carina and medial area steep anteriorly (Fig. 60, 65); second tergite smooth (including deep oblique anterior grooves) except strong median carina connected to minute triangular medio-basal area, antero-lateral triangular areas large (Fig. 60); second metasomal suture strongly crenulated, laterally narrowed and weakly up curved; medially second tergite 1.5 times longer than third tergite; maximum width of third tergite 2.7 times its medial length; third and fourth tergites with rather weakly median carina; third–fifth tergites smooth and without transverse subposterior groove and with long antero-lateral grooves; ovipositor sheath 0.63 times as long as fore wing and 0.65 times body; hypopygium ending just anterior of apex of metasoma (Fig. 55).

*Colour*. Yellowish brown; antenna (included scapus) and mandible apically dark brown; ovipositor sheath blackish with yellow setae; stigmal spot rather small, up to 0.4 anterior of first discal cell (Fig. 56); apical 0.2 of pterostigma dark brown and remainder yellow; veins yellow except dark brown basal half of 1-SR+M and most of 1-SR; wing membrane yellow, except for stigmal spot and slightly infuscate apical area of fore wing medium-sized, but remaining far from vein 1r-m (Figs 56, 57).

##### Distribution.

*China (Yunnan).

##### Etymology.

Named in honour of Prof. Dr Jun-hua He (Hangzhou) for his significant contribution to our knowledge of the Chinese Hymenoptera.

#### Maculibracon luteonervis sp. n.

Taxon classificationAnimaliaHymenopteraBraconidae

http://zoobank.org/1C7553C2-DF16-437F-AC89-95E3BBF141EC

Figs 67 , 68–79

##### Type material.

Holotype, ♀ (IZAS), “[**China**:] Yunnan, Xishuangbanna, Menghun, 750 m, 1.VI.1958, Yiran Zhang, No. IOZ(E)1964632”.

##### Diagnosis.

Scapus mainly yellowish brown, except for dark brown stripe on outer side, rather slender and less protruding ventrally (Fig. 74); head roundly narrowed posteriorly (Fig. 76); propodeum medio-posteriorly with smooth protuberance in lateral view (Fig. 78); medial area of first tergite low anteriorly (Fig. 78); wing membrane yellow with slightly infuscate apical area of fore wing wide and rather close to vein 1r-m; stigmal spot of fore wing up to anterior half of first discal cell, enclosing 0.6 of vein 1-SR+M (Figs 68, 69); pterostigma entirely yellow (Fig. 68); medio-basal area of second tergite nearly rhombic (Fig. 72); vein 1-SR+M of fore wing yellow; body and hind leg brownish yellow; length of ovipositor sheath 0.8 times fore wing and 0.8 times body. Similar to *Maculibracon laertius* (Cameron, 1903) because of the yellowish scapus in dorsal view, medium-sized stigmal sport of the fore wing, anteriorly low medial area of the first tergite and the wide apical infuscate area of the fore wing. The new species differs by having the apex of the pterostigma yellow (*versus* dark brown in *Maculibracon laertius*), ovipositor sheath 0.8 times fore wing (*versus* about 0.4 times), stigmal spot nearly square (*versus* obliquely narrowed) and head gradually roundly narrowed posteriorly in dorsal view (*versus* obliquely narrowed).

##### Description.

Holotype, ♀, length of body 17.1 mm, of fore wing 16.7 mm, of ovipositor sheath 13.0 mm.

*Head*. Antenna incomplete, left antenna with 75 segments remaining; scapus rather stout, 1.5 times longer than wide and distinctly emarginated apically, slightly longer ventrally than dorsally in lateral view, with a narrow apical ledge at inner side and gradually narrowed basally (Fig. 74); third and fourth segments 1.4 and 1.0 times their maximum width, respectively; length of maxillary palp as long as the height of head; inner side of eye not emarginated but slightly sinuate (Fig. 75); face moderately convex but flattened medially and medio-dorsally with weak median crest, densely punctate, and with long erect setae; clypeus flat, punctate, dorsally with coarse curved carina and ventral margin thin and lamelliform, with few long setae ventrally; hypoclypeal depression 0.4 times as wide as minimum width of face (Fig. 75); frons shallowly concave behind antennal sockets, smooth, except for a rather shallow median groove (Fig. 75); vertex smooth, with few long setae and shiny; stemmaticum distinctly protruding; OOL:diameter of posterior ocellus:POL = 24:9:8; in dorsal view length of eye 1.9 times temple; temples gradually roundly narrowed behind eyes, with some long setae, almost smooth dorsally and sparsely punctate ventrally (Figs 76, 77); malar suture shallow, curved; length of malar space 0.8 times basal width of mandible; mandible twisted, both teeth robust and subequal.

*Mesosoma*. Length of mesosoma 2.1 times its height (Fig. 70); side of pronotum shiny and smooth; propleuron with spaced punctures; pronotum vertical anteriorly, with a shallow pronope and groove and narrow antescutal depression; mesopleuron smooth and glabrous, except for an oblique band with punctures and short setae; mesosternal sulcus smooth and narrow; metapleuron smooth and with long setae, convex; mesoscutum glabrous except some setae near notaulic courses, shiny and smooth; notauli smooth, only distinct anteriorly and medially shallowly impressed; scutellar sulcus present and with distinct fine crenulae; scutellum distinctly convex and smooth except for some punctures; side of scutellum smooth; metanotum medio-anteriorly with short carina, posteriorly evenly convex and smooth; propodeum distinctly remotely punctate, with long setae, evenly convex but medio-posteriorly with one smooth protuberance (Fig. 71), ribbed in lateral view (Fig. 78).

*Wings*. Fore wing (Fig. 68): m-cu 0.8 times as long as 1-M; 1-SR+M angularly bent subbasally 1.4 times as long as 1-M; 3-SR weakly curved, and SR1 straight; r:3-SR:SR1 = 10:55:59; 2-SR:3-SR:r-m = 20:55:19; r-m largely sclerotised; 1-CU1 slightly widened and 0.18 times 2-CU1; cu-a weakly inclivous; CU1b nearly as wide as 3-CU1. Hind wing (Fig. 69): with 6 coarse subbasal bristles on C+SC+R and with 3 hamuli on R1; SR weakly curved basally and marginal cell parallel-sided apically; area near cu-a glabrous; 1r-m straight and 0.95 times as long as SC+R1; 2-SC+R twice longer than wide.

*Legs*. Tarsal claws simple and with long bristly setae ventrally; length of femur, tibia and basitarsus of hind leg 5.3, 10.0 and 7.9 times their maximum width, respectively; hind tibia with dense and rather appressed setae; one hind tibial spur 0.2 times as long as hind basitarsus, the other is broken; inner side of hind tibia and tarsus densely bristly setose (Fig. 73).

*Metasoma*. Length of first tergite 1.5 times its apical width, dorso-lateral carinae strongly developed, medial area smooth except for high median carina and medial area steep anteriorly (Figs 72, 78); second tergite smooth (including deep oblique anterior grooves) except strong median carina connected to minute nearly rhombic medio-basal area, antero-lateral triangular areas large (Fig. 72); second metasomal suture strongly crenulate, laterally narrowed and weakly up curved; medially second tergite 1.4 times longer than third tergite; maximum width of third tergite 2.7 times its medial length; third–fifth tergites smooth and without transverse subposterior groove and with long antero-lateral grooves; ovipositor sheath 0.78 times as long as fore wing and 0.76 times body; hypopygium ending just anterior of apex of metasoma (Fig. 67).

*Colour*. Brownish yellow; antenna (scapus mainly yellowish brown, except for dark brown stripe on outer side) and mandible apically dark brown; ovipositor sheath blackish with yellow setae; stigmal spot medium-sized, up to anterior half of first discal cell, enclosing 0.6 of vein 1-SR+M (Fig. 68); veins yellow (included 1-SR+M) except dark brown basal half of 1-SR; pterostigma entirely yellow (Fig. 68); wing membrane yellow, with slightly infuscate apical area of fore wing wide and rather close to vein 1r-m (Figs 68, 69).

##### Distribution.

*China (Yunnan).

##### Etymology.

Named after the yellow vein 1-SR+M of the fore wing, contrasting with the dark brown surrounding stigmal spot. “Luteus” is Latin for “yellow” and “nervus” for “sinew, vein”.

#### Maculibracon simlaensis

Taxon classificationAnimaliaHymenopteraBraconidae

(Cameron, 1899) comb. n.

Figs 80 , 81–91

Bracon simlaensis Cameron, 1899: 65–66.
Iphiaulax simlaensis ; Baltazar 1972: 273 (lectotype designation); Shenefelt 1978: 1795.
Craspedolcus simlaensis ; Quicke 1985: 357; van Achterberg and O’Toole 1993: 37.
Bracon lepcha Cameron, 1899: 69–68.
Iphiaulax lepcha ; Baltazar 1972: 272; Shenefelt 1978: 1776.
Craspedolcus lepcha ; Quicke 1985: 356; van Achterberg and O’Toole 1993: 24 (as synonym of Craspedolcus simlaensis (Cameron, 1899)).
Iphiaulax bhotanensis Cameron, 1907: 4; Dover 1925: 40 (as synonym of Iphiaulax lepcha (Cameron, 1899); Shenefelt 1978: 1776. **Syn. n.**
Craspedolcus bhotanensis ; Quicke 1985: 357.
Ipobracon maculicosta Enderlein, 1920: 71–72; Shenefelt 1978: 1824. **Syn. n.**
Craspedolcus maculicosta ; Quicke and van Achterberg 1990: 252, 259 (lectotype designation).

##### Material.

(2 ♀; IZCAS): 1 ♀, “[**China**:] Hainan, Ledong, 26.VIII.1984, Zhiqing Chen, No. IOZ(E)1964588”; 1 ♀, “Hainan, Jianfengling, 13.V.1984, Maobin Gu, No. IOZ(E)1964589”; 1 ♀ (RMNH), “C. **Vietnam**: Thua Thien Hué, Phong Dién N. R., n[ea]r base-camp, 50-100 m, 25.iii.2001, C. v. Achterberg, RMNH’01”.

##### Diagnosis.

Scapus mainly yellowish brown, except for dark brown stripe on outer side, rather slender, twice as long as wide and less protruding ventrally (Fig. 91); head less narrowed posteriorly (Fig. 88); propodeum medio-posteriorly with smooth protuberance in lateral view (Fig. 90); wing membrane yellow except wide and slightly infuscate apical area of fore wing, rather close to vein 1r-m; stigmal spot of fore wing up to vein m-cu, enclosing nearly entire vein 1-SR+M (Figs 81, 82); vein 1-SR+M of fore wing dark brown; pterostigma narrowly blackish apically and remainder yellow (Fig. 81); medial area of first tergite low anteriorly (Figs 85, 90); medio-basal area of second tergite distinctly triangular (Fig. 85); body and hind leg brownish yellow; length of ovipositor sheath 0.7 times fore wing and 0.7 times body.

*Variation*. Length of body of female 16.7–19.2 mm, of fore wing of female 16.3–18.5 mm, and of ovipositor sheath 11.2–12.5 mm; antenna of female with 83 (1), 95 (1) segments; apical antennal segment with short spine; penultimate segment 1.1–1.2 times their maximum width; vein 3-SR of fore wing 2.4–2.5 times vein 2-SR; length of first tergite 1.4–1.7 times its apical width; length of ovipositor sheath 0.68–0.69 times fore wing; mesosoma and metasoma ventrally yellowish brown or infuscated.

##### Distribution.

India, Bhutan, Myanmar, Indonesia (Java), *Vietnam, *China (Hainan).

## Supplementary Material

XML Treatment for Craspedolcus

XML Treatment for Craspedolcus fraternus

XML Treatment for Craspedolcus politus

XML Treatment for Maculibracon

XML Treatment for Maculibracon abruptus

XML Treatment for Maculibracon hei

XML Treatment for Maculibracon luteonervis

XML Treatment for Maculibracon simlaensis
